# Epigenetic Modification of Cytosines in Hematopoietic Differentiation and Malignant Transformation

**DOI:** 10.3390/ijms24021727

**Published:** 2023-01-15

**Authors:** Jungeun An, Myunggon Ko

**Affiliations:** 1 Department of Life Sciences, Jeonbuk National University, Jeonju 54896, Republic of Korea; 2 Department of Biological Sciences, Ulsan National Institute of Science and Technology, Ulsan 44919, Republic of Korea; 3 Center for Genomic Integrity, Institute for Basic Science, Ulsan 44919, Republic of Korea

**Keywords:** CpG methylation, DNMT enzyme, TET dioxygenases, hematopoietic stem cells, clonal hematopoiesis, blood cancer

## Abstract

The mammalian DNA methylation landscape is established and maintained by the combined activities of the two key epigenetic modifiers, DNA methyltransferases (DNMT) and Ten-eleven-translocation (TET) enzymes. Once DNMTs produce 5-methylcytosine (5mC), TET proteins fine-tune the DNA methylation status by consecutively oxidizing 5mC to 5-hydroxymethylcytosine (5hmC) and further oxidized derivatives. The 5mC and oxidized methylcytosines are essential for the maintenance of cellular identity and function during differentiation. Cytosine modifications with DNMT and TET enzymes exert pleiotropic effects on various aspects of hematopoiesis, including self-renewal of hematopoietic stem/progenitor cells (HSPCs), lineage determination, differentiation, and function. Under pathological conditions, these enzymes are frequently dysregulated, leading to loss of function. In particular, the loss of DNMT3A and TET2 function is conspicuous in diverse hematological disorders, including myeloid and lymphoid malignancies, and causally related to clonal hematopoiesis and malignant transformation. Here, we update recent advances in understanding how the maintenance of DNA methylation homeostasis by DNMT and TET proteins influences normal hematopoiesis and malignant transformation, highlighting the potential impact of DNMT3A and TET2 dysregulation on clonal dominance and evolution of pre-leukemic stem cells to full-blown malignancies. Clarification of the normal and pathological functions of DNA-modifying epigenetic regulators will be crucial to future innovations in epigenetic therapies for treating hematological disorders.

## 1. Introduction

Methylation of cytosine residue in a cytosine-guanine (CpG) dinucleotide is an extensively studied epigenetic mechanism that is catalyzed by DNA methyltransferases (DNMTs), yielding 5-methylcytosine (5mC), the fifth base in DNA. Cytosine methylation serves as a conserved epigenetic mark and exerts profound effects on a spectrum of fundamental processes in cells, including DNA–protein interaction, transcription, chromatin architecture and stability, chromosome segregation, and the integrity of the genome [1,2,3]. As a result, CpG methylation has important implications for key biological processes, including long-term monoallelic repressions such as X chromosome inactivation and genomic imprinting, as well as the silencing of endogenous parasitic sequences (i.e., retrotransposons) and tumor-suppressor genes. In general, high levels of 5mC at promoters can be associated with transcriptional silencing, although their correlation at the genome-wide level is low [1,4]. Promoter methylation can repress transcription by facilitating the formation of a transcriptional repressor complex via the recruitment of 5mC-recognizing proteins such as methyl-CpG-binding proteins (MBDs) or by directly blocking the binding of transcription factors (TFs) [5,6,7,8]. In contrast, gene body methylation tends to be positively correlated with gene transcription [9]. Besides normal biology, DNA methylation is dysregulated under pathological conditions, critically impacting a variety of processes, including every stage of cancer development (i.e., initiation, maintenance, and progression). Indeed, the DNA methylation pattern is recurrently perturbed in cancer and is thus considered a classic hallmark of cancer. Cancer genomes generally display two characteristic patterns of aberrant DNA methylation: a focal increase in DNA methylation at gene promoters (associated with transcriptional silencing of key tumor-suppressor or repair genes) and a global reduction in DNA methylation across the genome (associated with activation of parasitic sequences and genomic instability).

**Figure 1:**
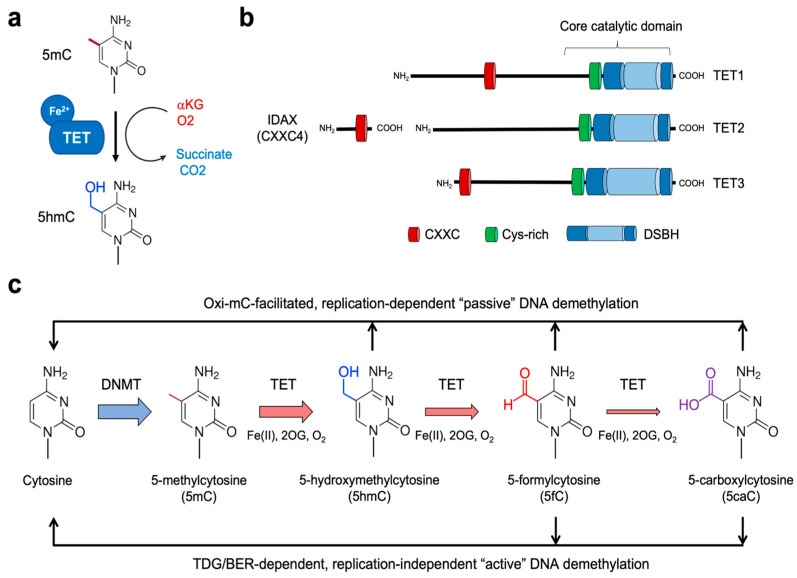
Role of TET proteins in 5mC oxidation and DNA demethylation. (**a**) TET proteins belong to the family of Fe^2+^- and αKG-dependent dioxygenases that oxidize their substrates. All three TET family members successively oxidize 5mC to 5hmC, 5fC, and 5caC. (**b**) Proteins of the TET family consist of TET1, TET2, and TET3. All three TET proteins have highly conserved catalytic domains at the carboxyl-terminal region, which is composed of the Cys-rich (Cys) and double-stranded β-helix (DSBH) domain. While TET1 and TET3 have the CXXC domain at their amino-terminal regions, TET2 does not contain it. Instead, during evolution, the chromosomal inversion separated the region encoding the CXXC domain of primordial TET2 from that encoding its catalytic domain, giving rise to a unique gene *IDAX* (which is also called *CXXC4*). (**c**) DNMTs methylate cytosine to yield 5mC, which is further oxidized by TET proteins. The oxidized methylcytosines (called oxi-mCs) interfere with DNMT1, thus promoting “passive” DNA demethylation after replication. Moreover, 5fC and 5caC are recognized and cleaved by the DNA repair protein TDG. Then, the resultant abasic sites are repaired by the base-excision repair (BER) pathway, a process called replication-independent “active” DNA demethylation.

5mC has been considered a very stable base since its discovery. Thus, cytosines were initially thought to exist in either methylated or unmethylated states [10]. However, a further layer of complexity to the covalent modification of cytosine has been revealed as we understood the function of the TET family of dioxygenases. TET proteins fine-tune cytosine methylation by oxidizing the methyl group of 5mC to form 5-hydroxymethylcytosine (5hmC), a process termed DNA hydroxymethylation (Figure 1a). They can further oxidize the hydroxyl group of 5hmC to generate 5-formylcytosine (5fC) and 5-carboxylcytosine (5caC). Notably, TET-mediated oxidation of 5mC up to these higher oxidation states (5fC and 5caC) provides routes to the activation of replication-independent demethylation (which will be discussed in detail later). Together, the methylome landscape in the mammalian genome is exquisitely regulated by the complex interplay between DNMT and TET activities.

Recent large-scale sequencing analyses have successfully revealed a comprehensive catalog of mutational signatures in a wide variety of cancers, thereby facilitating the identification and functional characterization of candidate cancer-causing driver mutations [11,12,13,14]. In hematological neoplasms, numerous somatic mutations recurrently occur in the genes encoding various epigenetic modifiers, including histone/DNA modification enzymes and chromatin remodelers. *DNMT3A* and *TET* genes are among the genes most frequently mutated in clonal hematopoiesis and hematologic cancers. Thus, in this review, we briefly review recent progress in our understanding of how both enzymes contribute to the DNA methylation homeostasis, normal hematopoiesis, and malignant transformation, focusing our discussion on the potential molecular mechanisms underlying hematological oncogenesis driven by DNMT3A and TET2 dysregulation.

## 2. Maintenance of Cytosine Methylation Homeostasis by DNMT and TET Proteins

### 2.1. Establishing and Maintaining the Mammalian Methylation Landscape

Epigenetic modifications imposed on the mammalian genome confer stability and diversity to the functional state of cells by creating chemically stable but reversible marks that have a direct effect on the local gene activity. DNA cytosine methylation is a central epigenetic modification that is faithfully inherited from parent to daughter cells, a feature that is critical for the preservation of specific gene expression programs and cellular identity across cell divisions [15,16]. On the other hand, DNA methylation is highly mutagenic as 5mC undergoes rapid deamination to thymine, causing C-to-T transition [17]. This inherent mutability of methylated cytosines results in a much lower frequency of the CpG dinucleotides (3~8% of all cytosines) in the genome than expected while increasing a natural source of genetic variations to facilitate the emergence of novel heritable epimutations and epialleles. 

Enzymes of the DNMT family are the “writers” of cytosine methylation that catalytically remove a methyl group (−CH_3_) from S-adenosylmethionine (SAM) and put it at the 5-position (C5) of cytosine to yield 5mC [18,19,20] (Figure 1). Although the mammalian genome shows profound asymmetry in terms of the distribution of CpG-rich and CpG-poor regions, DNMTs typically catalyze symmetrical methylation of cytosine in a 5′-CpG-3′ dinucleotide on both strands of DNA [18,19,20]. DNA methylation patterns are relatively stable in most cell types [21], with over 80% of CpG sites being methylated [22]. However, a small fraction of CpG sites is variably methylated in different tissue lineages and predominantly co-localize with distal *cis*-regulatory elements (CREs), particularly enhancers and TF binding sites [23].

During early embryogenesis, the *de novo* DNA methyltransferases DNMT3A and DNMT3B initially deposit the methylation marks on unmethylated templates. Once established, the canonical maintenance methyltransferase DNMT1 ensures the somatic inheritance of the pre-existing methylation patterns via post-replicative methylation of the nascent DNA strand. During the S phase of the cell cycle, DNA replication machinery does not copy 5mC on the parental strand onto the newly synthesized daughter strand, resulting in hemimethylated DNAs. Then, DNMT1 localized to the replication fork restores the symmetrical methylation by methylating the nascent strand. DNMT1 has a marked preference for hemimethylated CpGs due to its physical association with the ubiquitin-like plant homeodomain and RING finger domain-containing protein 1 (UHRF1; also known as NP95) and the proliferating cell nuclear antigen (PCNA) [24,25,26,27,28]. The vast majority of hemimethylated CpG sites are methylated very rapidly within 20 min of replication, although a small fraction of them remain stably hemimethylated and are inherited at CCCTC-binding factor (CTCF)/cohesin-binding sites that regulate chromatin assembly [29]. The failure of maintenance methylation due to impaired expression or function of DNMTs results in replication-dependent “passive” demethylation. 

The long-standing view on the divergent functions of DNMT family members as either maintenance (DNMT1) or *de novo* (DNMT3A/3B) methylases, respectively, has recently been challenged. DNMT1 has been shown to possess a *de novo* methyltransferase activity in vitro and in vivo [30,31], which is particularly important for the stable repression of retrotransposons [31]. Moreover, DNMT1 alone is not capable of handling maintenance methylation entirely [32,33]. Intriguingly, DNMT3A and DNMT3B exhibit similar activities toward unmethylated and hemimethylated DNA in vitro and can contribute to the maintenance methylation in many cells, including mouse embryonic stem cells (ESCs), neuronal cells, and hematopoietic cells [32,34,35,36,37]. In the absence of DNMT3A/3B, ESCs show high levels of hemimethylated DNA (~30% of CpG sites) in the repetitive elements [38]. These results suggest that the maintenance of mammalian DNA methylome relies on the combined activities of all three DNMTs: the predominant DNA methylase DNMT1 catalyzes the bulk of methylation at the replication forks, particularly on the hemimethylated DNA in dividing cells, and DNMT3A/3B catalyze ongoing methylation of newly replicated CpG sites to complete methylation at specific chromatin regions such as repeat sequences [39].

### 2.2. Iterative Oxidation of 5mC and DNA Demethylation by TET Proteins

Until recently, 5mC has been considered a terminal cytosine modification form that either remains a stable base or reverts to cytosine through demethylation. Besides the (replication-dependent) passive demethylation, the dynamicity of 5mC abundance is also controlled by the (replication-independent) active demethylation pathway [40,41]. In mammals, the mechanism underpinning passive demethylation is relatively well elucidated. However, it had remained a mystery how 5mC was actively reversed independently of DNA replication until the landmark discovery of the TET enzyme function as a 5mC oxidase [42]. 

The TET family of dioxygenases, including TET1, TET2, and TET3, directly influence the methylation states by serving as the 5mC “erasers” (Figure 1b) [42,43,44,45]. In 2009, the Rao group discovered the function of TET1 protein based on its homology to base J-binding proteins (JBPs), the thymidine hydroxylases that catalyze the first step in the biosynthesis of an unusual base called base J (β-d-glucosyl-hydroxymethyl-uracil) in kinetoplastid DNAs [42,46,47]. TET proteins belong to the Fe^2+^- and α-ketoglutarate (αKG, also known as 2-oxoglutarate)-dependent dioxygenase family [48]. Unlike thymidine hydroxylases that oxidize thymine, TET proteins catalyze in situ hydroxylation of 5mC in the 5mCpG dinucleotide to yield 5hmC through an oxidation reaction requiring molecular oxygen, reduced iron (Fe^2+^), and tricarboxylic-acid-cycle intermediate αKG (Figure 1a). They first transfer a hydroxyl group (−OH) to the methyl group of 5mC to generate 5hmC by transferring one atom of molecular oxygen to the C5-methyl group of 5mC; meanwhile, αKG undergoes oxidative decarboxylation by the other oxygenic atom, releasing CO_2_ and succinate as byproducts (Figure 1a) [49]. TET proteins carry out two additional oxidation reactions to sequentially oxidize 5hmC to 5-formylcytosine (5fC) and 5-carboxylcytosine (5caC) [42,44,45]. All TET members enable successive 5mC oxidation due to the conserved catalytic core domains at their carboxyl-terminal regions (Figure 1b). In line with their strong functional link to DNA methylation, TET orthologues are strictly restricted to metazoan organisms that possess cytosine methylation machinery [47,48]. It has been shown that 5fC and 5caC are produced by iterative actions of TET2 protein in a single encounter with 5mC-containing DNA without releasing 5hmC intermediates and that this catalysis is not significantly affected by the modification status of the complementary CpG sites [50]. 

All three forms of oxidized methylcytosines (oxi-mCs) play a vital role in all known pathways of DNA demethylation in mammals (Figure 1c) [51,52]. First, oxi-mCs in the template strand impair maintenance methylation by interfering with the DNMT1/UHRF1 complex. Thus, the original DNA methylation is lost after cell division [53,54] unless it is maintained by the other methylases DNMT3A/3B. This oxi-mC-facilitated passive demethylation is considered the principal mechanism for the priming of the promoters or enhancers of lineage-specifying genes in dividing cells (Figure 1c) [55,56,57]. Second, replication-independent 5mC removal primarily implicates TET-mediated 5mC oxidation up to 5fC and 5caC, which are removed from the DNA backbone by the DNA repair enzyme thymine DNA glycosylase (TDG) (Figure 1c). TDG typically remove thymine from T:G mismatches and can also hydrolyze the glycosidic linkage between the sugar moiety of DNA and 5fC/5caC that normally pair with G. This cleavage results in abasic sites that are eventually repaired to unmodified cytosine through a base excision repair (BER) pathway [44,58,59,60]. Thus, TET-catalyzed oxi-mCs are pivotal intermediates in active DNA demethylation. 

In addition to its role in maintaining enhancer activity to promote cell fate determination, TET-mediated active DNA demethylation was recently shown to generate endogenous DNA damage, particularly single-strand DNA breaks during the BER process [61]. In many cell types, TET-dependent active demethylation seems to play a minor role in replicating cells compared with passive demethylation. Interestingly, TET2-mediated 5mC oxidation was stalled at 5hmC when a conserved residue (Thr1372) in its active site was mutated [62]. As TDG-dependent active demethylation requires oxidation up to 5fC and 5caC, this TET2 variant would be useful to evaluate to what extent TET-mediated active demethylation contributes to certain cellular processes. Furthermore, oxi-mC intermediates have roles as unique epigenetic marks independently of the DNA demethylation pathway, presumably by influencing the chromatin association of methyl CpG-binding proteins or specific oxi-mC-interacting proteins or other epigenetic mechanisms. Thus, it is also useful to probe the biological functions of 5hmC separately from further oxidation products.

Alternatively, AID/APOBEC family enzymes are shown to deaminate 5mC or 5hmC to uracil or 5-hydroxymethyl-uracil, respectively, which are subsequently reverted to cytosine by BER enzymes [63,64]. TDG seems to act as a common mediator in the various demethylation pathways, and its deficiency indeed disrupts normal methylation patterns [65]. However, the active demethylation in the zygotic genome remains unaffected even in the absence of TDG [66], suggesting that there might be unidentified additional strategies by which cells accomplish active demethylation independent of the TET/TDG-dependent pathway. As a potential mechanism, mouse ESCs are shown to possess a 5caC decarboxylase activity [67], although the responsible enzyme remains to be identified.

## 3. Epigenetic Regulation of Clonal Hematopoiesis by DNMT and TET Proteins

**Figure 2:**
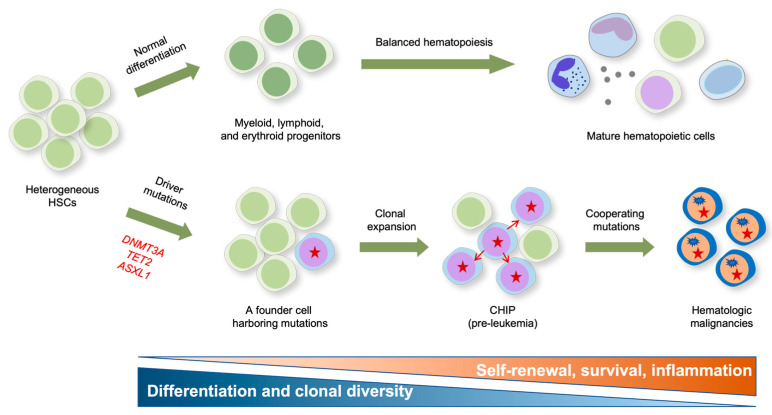
Impact of epigenetic mutations on the formation and clonal evolution of pre-leukemic stem cells to hematologic malignancies. A model for clonal expansion of hematopoietic stem cells (HSCs) to establish the preleukemic condition that eventually evolves into frank malignancies is shown. Healthy HSCs self-renew and differentiate into multipotent progenitors that give rise to functional tri-lineage hematopoietic cells in the periphery (*upper panel*). However, aging HSCs acquire somatic mutations in genes encoding the key epigenetic modifiers *DNMT3A* and *TET2* that confer competitive fitness advantage and drive the clonal expansion of mutant HSCs, resulting in CHIP (*lower panel*). *DNMT3A* mutation is presumed to mainly influence multipotency (i.e., self-renewal), and *TET2* mutations preferentially influence inflammation and myeloid bias, respectively. The pre-leukemic stem cells eventually evolve into full-blown malignancies after acquiring subsequent cooperating mutations in genes encoding *FLT3, ASXL1, JAK2, EZH2, NRAS, KIT, RHOA, DNMT3A, SRSF2, AML-ETO1*, etc.

Clonal hematopoiesis of indeterminate potential (CHIP) refers to the aberrant expansion of hematopoietic cell clones without overt abnormalities such as cytopenia, dysplasia, or neoplasia [68,69,70]. CHIP arises from competition over a long period among long-lived HSCs in the bone marrow. Large cohort studies in humans with advanced age have identified ~20 somatic mutations as potential cell-intrinsic contributors to clonal dominance in CHIP. Most of these mutations typically fall within the three functional categories, including epigenetic modifiers (e.g., *DNMT3A*, *TET2*, and *ASXL1*), splicing factors (e.g., *SF3B1* and *SRSF2*), and regulators of DNA damage response (*PPM1D* and *TP53*) [69]. In particular, somatic mutations in epigenetic modifiers are remarkably widespread, with ~70% of all CHIP-associated variants being mutations in *DNMT3A*, *TET2*, and *ASXL1* (Figure 2). 

Different CHIP mutations are shown to drive clonal expansion with substantially different growth rates, and mutations driving faster clonal growth tend to carry an increased risk of malignancy [71]. While *DNMT3A* mutant clones preferentially expanded early in life and underwent a slower clonal expansion in old age, *TET2* mutations emerged across all ages and induced faster clonal expansion, resulting in TET2 becoming the most prevalent CHIP driver in old age [71]. Consistent with their potential roles in CHIP, *DNMT3A* and *TET2* mutations are early events occurring in HSCs during the clonal evolution to leukemia (Figure 2) [72,73]. Notably, *DNMT3A* mutations occur more frequently in multipotent HSCs and propagate in all blood lineages, while *TET2* mutations occur in more committed progenitors with myeloid potential, suggesting that *DNMT3A* mutations primarily contribute to multipotency and that *TET2* mutations confer a strong myeloid bias [74]. To functionally characterize these variants, many murine models harboring a conditional deletion of these genes in a hematopoietic system have been generated. These animal models show that mutations augmenting HSC self-renewal and fitness, rather than those influencing their balanced differentiation, exert the most potent effect on CHIP in general [75]. In particular, the LOF of DNA methylation regulators DNMT3A and TET2 efficiently drives CHIP by rendering the HSPC more competitive through enhanced self-renewal and restricted differentiation, as described below. 

## 4. DNMTs in Normal and Malignant Hematopoiesis

### 4.1. DNMTs in HSC Self-Renewal and Lineage Specification

CpG methylation stabilizes the self-renewal and lineage commitment of HSPCs during normal hematopoiesis [76,77,78,79]. Many knockout (KO) studies in mice have demonstrated that constitutive methylation is essential for the maintenance of stemness (i.e., self-renewal and multipotency)-related gene expression as well as the suppression of premature activation of lineage-affiliated genes in HSPCs (Table 1). Despite identical biochemical activities, different DNMT family members exert distinct effects on these processes, although the reason for this is not understood entirely. Loss of DNMT1 in mice, as achieved by conditional gene deletion or the expression of a hypomorphic variant in the DNMT1 KO background, significantly disrupted the homeostasis and self-renewal of HSCs regardless of transplantation stress [76,79]. DNMT1 deficiency led to DNA hypomethylation in HSCs, resulting in widespread transcriptional deregulation. Strikingly, these transcriptional alterations occurred in a lineage-specific manner: myeloerythroid genes (e.g., *Gata1*, *Id2,* and *Cebpa*) were derepressed, whereas lymphoid and stem cell-related genes were downregulated in HSCs, which was supported functionally by markedly skewed differentiation toward myeloerythroid lineages with impaired lymphopoiesis [76]. These observations support the notion that DNA methylation may epigenetically divert lymphoid progenitors from the default differentiation program toward a myeloid lineage. 

On the other hand, consistent with the recurrent *DNMT3A* mutation in CHIP, DNMT3A loss in mice augmented the self-renewal of HSCs, with their differentiation being compromised over serial transplantation on a per-HSC basis (i.e., a lower output of mature blood cells per HSC) [77,80,81]. Compared with DNMT3A loss, DNMT3B LOF displayed similar but milder phenotypes, and simultaneous deletion of DNMT3A and DNMT3B exhibited synergistic effects, causing enhanced HSC self-renewal and a more severe differentiation block [80]. While it remains to be elucidated how DNMT3A loss exerts this profound effect on HSCs, several pieces of evidence suggest that modulation of DNA methylation by DNMT3A at HSC regulatory elements plays a critical role. In DNMT3A-KO HSCs, overall methylation levels remained largely unchanged, and even alterations in methylation were poorly correlated with changes in gene expression in general. However, DNMT3A deficiency seemed to induce notable hypomethylation and derepression of the key multipotency-related genes, including *Runx1, Gata3, Pbx1*, and *Cdkn1a*, while downregulating differentiation-associated genes such as *Flk2, Ikaros, Sfpi1* (*Pu.1*), and *Mef2c*. Intriguingly, DNMT3A loss allowed HSCs to self-renew over at least 12 rounds of transplantation by reducing DNA methylation at enhancers or canyons associated with self-renewal genes [82]. Thus, upon differentiation signals, DNMT3A may methylate and repress a handful of HSC self-renewal genes to allow for downstream differentiation.

### 4.2. Dysregulation of DNMT3A in Hematologic Malignancies

The DNA methylation abnormalities arising from dysfunctional DNMTs are linked to the initiation and progression of hematological cancers. Unlike *DNMT1*, somatic mutations in *DNMT3A* are prevalent in hematologic malignancies of myeloid and lymphoid lineage, including AML (~20%) and myelodysplastic syndromes (MDS; ~10%), and these mutations are associated with a poor prognosis [83]. In AML, *DNMT3A* mutations are highly enriched for heterozygous point mutations at position R882 (most commonly R882H) within the catalytic domain. The *DNMT3A^R882^* hotspot mutation is a hypomorph that diminishes methyltransferase activity to ~20% of normal levels by disrupting active tetramer formation, thus acting in a dominant negative manner [84]. In addition, multiple nonsense or frameshift mutations are also presumed to produce truncated forms of the DNMT3A enzyme with defective methylase activity [85]. Notably, most CHIP-related *DNMT3A* mutations are also heterozygous and presumed to be LOF mutations, and these mutations occur all along the length of the gene, although R882 mutations are also frequent [70]. 

**Table 1:** Hematopoietic phenotypes of DNMT-deficient animal models.

Genotype	Major KO Mice Phenotype	Hematologic Malignancy	References
*Dnmt1^−/chip^*	Disrupted HSC homeostasis and self-renewal; diminished repopulation capacity; myeloerythroid skewing; derepression of myeloerythroid genes and suppression of lymphoid and stem cell-related genes in HSCs	Not observed	[76]
*Dnmt1^fl/fl^ Mx1-Cre*	Defective HSC self-renewal, BM niche retention, and multilineage differentiation; diminished repopulation capacity; enhanced myeloid lineage gene expression	Not observed	[79]
*Dnmt3a^fl/fl^ Mx1-Cre* (competitive transplantation)	Augmented HSC self-renewal and suppressed differentiation over serial transplantation; global hypomethylation and CpG island hypermethylation; increased expression of multipotency genes but reduced expression of differentiation genes in HSCs	Not observed	[77]
*Dnmt3a^fl/fl^ Dnmt3b^fl/fl^ Mx1-Cre* (competitive transplantation)	Similar but milder effect in *Dnmt3b* KO mice; synergistic effects of double KO in enhancing HSC self-renewal; mild global hypomethylation; HSC differentiation block due to activated β-catenin signaling	Not observed	[80]
*Dnmt3a^fl/fl^ Tet2^fl/fl^ Mx1-Cre* (competitive transplantation)	Limitless self-renewal of *Dnmt3a* KO HSC in vivo; exhaustion of *Tet2* KO HSC; myeloid skewing and rapid expansion of *Tet2* KO progenitors	Not observed	[81]
*Dnmt3a^fl/fl^ Mx1-Cre* (competitive transplantation)	Limitless self-renewal of *Dnmt3a* KO HSC in vivo (>12 rounds of transplantation); focal loss of DNA methylation at self-renewal-associated genes; compromised differentiation potential	Not observed	[82]
*Dnmt3a^+/−^*	Age-associated myeloid skewing and a competitive transplantation advantage	Myeloid malignancy (37.2% of mice at >20 mo; transplantable); no T cell leukemia	[85]
*EμSRα-tTA;Teto-Cre; Dnmt3a^fl/fl^*	Splenomegaly largely due to expansion of mature B1 B-cells; ~20% decrease in overall gene body methylation; hypomethylation of repetitive elements; CLL and T-cell malignancies in *Dnmt3a/b* double KO mice	Chronic lymphocytic leukemia (100%, median survival, 371 days, B-cell malignancy); no myeloid malignancy	[86]
*Dnmt3a^fl/fl^ Mx1-Cre* (non-competitive transplantation)	Lineage-specific methylation aberrations; acquisition of spontaneous mutations, including *Kras*; accelerated Nras-driven neoplasia by DNMT3A loss	Myeloid malignancy (MDS (24.39%), AML (19.51%); B-ALL and T-ALL (9.75%); median survival, 321 days	[87]
*Dnmt3a^fl/fl^ Mx1-Cre* (non-competitive transplantation)	Bone marrow failure; enhanced HSC serial replating capacity; dysfunctional myeloid and erythroid development; acquisition of *c-Kit* mutation	MDS-like disease (76%, median survival, 328 days; transplantable); MPD (16%) and AML (8%); cooperation with c-Kit mutation in the development of acute leukemia (median survival, 67 days)	[88]
*Dnmt3a^fl/fl^ Mx1-Cre*	Increased HSCPC self-renewal; cytopenia; impaired erythropoiesis; myeloproliferation	MDS/MPN (median survival, 48.6 wk; transplantable)	[89]
Tetracycline-inducible *Dnmt3b* knock-in	Impaired leukemia development and leukemia stem cell function; widespread DNA hypermethylation;	Blockade of Myc-Blc2- or MLL-AF9-induced leukemogenesis	[90]
BMT after retroviral overexpression of DNMT3A^R882H^	Aberrant expression of hematopoiesis-related genes with corresponding changes in gene body methylation	CMML-like disease (100% of mice)	[91]

Animal studies have shown that the LOF of DNMT3A can drive the transformation from HSPCs to different malignancies (Table 1). DNMT3A loss was not enough to immediately trigger the transformation of murine hematopoietic cells, but its long-term ablation predisposes mice to develop heterogeneous malignancies [85,86,87,88], suggesting that *DNMT3A* mutations possess moderate leukemogenic potential in vivo. Mice heterozygous for germ-line deletion of the *Dnmt3a* allele showed myeloid skewing and a competitive transplantation advantage and eventually developed transplantable myeloid malignancies after a long latency [85]. Likewise, conditional deletion of *Dnmt3a* in the hematopoietic system also resulted in lethal, fully penetrant, and transplantable myeloproliferative neoplasms (MPNs) with a median survival of 48.6 weeks [89]. In a separate study, conditional deletion of *Dnmt3a* in stem/progenitor cells led to chronic lymphocytic leukemia (CLL) with a median survival of 371 days, which was accelerated by the combined deletion of *Dnmt3b* [86]. However, no myeloid malignancies were observed in these animals. In contrast, forced expression of *Dnmt3b* in mice significantly delayed leukemogenesis induced by either Myc-Bcl2 or MLL-AF9 [90]. 

When *Dnmt3a* KO HSCs were transplanted into lethally irradiated mice without healthy bone marrow cells, all the mice died within one year of a range of hematologic malignancies such as MDS, AML, and T- and B-cell acute lymphoblastic leukemia (Table 1), the diseases also frequently observed in patients with *DNMT3A* mutations, and the sick mice acquired a variety of cooperating mutations [87,88]. Furthermore, chimeric mice reconstituted with bone marrow cells overexpressing the DNMT3A^R882H^ mutant also developed myeloproliferation resembling CMML due to impaired gene expression and DNA methylation [88,91]. Most of the DNMT-disrupted murine models display alterations in the DNA methylation patterns and transcriptional programs, but it is unclear whether the altered methylome is directly attributed to the transcriptional changes and malignant transformation. As many genes related to HSC self-renewal or dysregulated in leukemia (e.g., *HoxA9*, *Meis1*, and *Evi1*) were under the control of large undermethylated domains termed “canyons” whose boundaries were eroded in the absence of DNMT3A [37], DNMT3A-mediated methylation of canyon borders may also contribute to the suppression of transformation. Furthermore, it also remains to be clarified how DNMT3A inactivation results in diverse types of malignancies. Given that different types of diseases in the same DNMT3A KO model display distinct lineage-specific methylation profiles [87], deficiency of DNMT3A may induce pre-leukemia, which then transform into different types of leukemia depending on additional hits. 

## 5. TET Proteins in Normal and Malignant Hematopoiesis

### 5.1. Impaired TET Expression or Function in Myeloid and Lymphoid Malignancies

Although genes encoding TET1 and TET3 are rarely mutated in hematopoietic diseases, *TET2* frequently undergoes somatic mutation, affecting both lymphoid and myeloid lineages [92,93,94]. *TET2* mutations are also the second most common mutations in CHIP. *TET2* mutations are distributed along the length of its coding region, and many missense mutations are relatively clustered in the catalytic domain, mostly resulting in the LOF of the enzyme. *TET2* mutations are prevalent in a range of myeloid malignancies, including AML (~23%), MDS (~25%), MPN (~13%), and CMML (~50%), and also in lymphoid malignancies, including T cell lymphoma (~11.9%) and B cell lymphoma (~2%) [92,93,94]. 

Particularly, *TET2* mutations are highly recurrent events in peripheral T-cell lymphoma (PTCL) such as angioimmunoblastic T cell lymphoma (AITL; 33~63%) and PTCL, not otherwise specified (PTCL-NOS; 20~36%) [95,96,97]. Based on the transcriptional profiles, AITL is a highly aggressive form of PTCL driven by malignant cells derived from follicular helper T (Tfh) cells, and *TET2* mutations are more common in a subgroup of PTCL-NOS displaying Tfh-like features. Thus, *TET2* mutations in PTCL are thought to be associated with Tfh differentiation [96]. In PTCL, particularly AITL, mutations in *RHOA*, *DNMT3A,* and *IDH2* genes are also common, and *TET2* mutations often co-exist with these mutations [97,98,99,100], consistent with a notion that *TET2* mutations may cause preleukemic conditions and require additional mutations to drive full-blown diseases. 

*TET2* mutations are also frequent in B-cell malignancies, particularly in diffuse large B-cell lymphoma (DLBCL; 6~12%), the most common type of non-Hodgkin’s lymphoma arising from germinal center B cells. Notably, in an assay to quantify 5hmC in the genome of patients with various hematologic malignancies, levels of 5hmC in a significant proportion of patients with wild-type (WT) *TET2* (and also WT *TET1* and *TET3*) were as low as those from patients with *TET2* mutations [43]. This suggests that TET proteins can be inactivated even without mutations in their coding region, presumably through impaired expression or function of TET mRNAs or proteins. The potential mechanisms are extensively reviewed in [92,93,94]. Importantly, future studies are necessary to resolve whether functional inactivation of TET proteins via non-mutational venues also contributes to CHIP.

### 5.2. Context-Dependent Function of TET1 and TET3

Accumulated evidence indicates that individual TET family members have distinct impacts on HSC self-renewal, lineage specification, and differentiation (Table 2). Dysregulation of specific members results in oncogenesis toward distinct types of malignancies. Despite low expression in hematopoietic tissues and rare mutations in hematologic neoplasms, TET1 is indispensable for normal and malignant hematopoiesis. TET1 can promote or antagonize transformation depending on the context. As observed in many solid cancers [101], TET1 acts as a tumor suppressor in B-cell malignancy [102]. In non-Hodgkin’s B cell lymphomas, such as DLBCL or follicular lymphoma (FL), *TET1* was epigenetically silenced through promoter hypermethylation. TET1 loss resulted in DNA hypermethylation in murine HSPCs and disrupted the expression of many genes implicated in B lineage specification, chromosomal maintenance, and DNA repair [102]. As a result, *Tet1* KO mice were predisposed to increased self-renewal, DNA damage accumulation, and lymphoid skewing, eventually developing B-cell lymphoma after a long latency. However, it remains to be determined whether TET1 loss induces lymphoid skewing by influencing transcriptional priming in HSCs and also why lymphoid lineage cells are specifically susceptible to TET1 LOF. Given that *TET1* mutations are rare in CHIP, even though they seem to increase HSC self-renewal, TET1 LOF occurring independently of mutations may be implicated in driving clonal hematopoiesis. 

In contrast, TET1 can also act as an oncogene during leukemogenesis, particularly in T-cell acute lymphoblastic leukemia (T-ALL). TET1 was directly activated by MLL fusion proteins and enhanced oncogenic transcriptional programs involving *HOXA9*, *MEIS1*, and *PBX3*, thus facilitating leukemogenesis [103,104]. Furthermore, TET1 expression was higher in human T-ALL cell lines and clinical samples [105,106] than in normal bone marrow or B-ALL samples. TET1 depletion significantly disrupted the proliferation of human T-ALL cells in vitro and in vivo by impairing the expression of many oncogenes and DNA repair genes [106]. Interestingly, the PARP inhibitor Olaparib substantially reduced TET1 expression and blocked the leukemic growth of T-ALL cells. In addition, increased *TET1* expression was associated with the poor survival of patients with cytogenetically normal acute myeloid leukemia (CN-AML) [107], suggesting that TET1 may play a role as an oncogene in AML. It remains to be fully elucidated how TET1 exerts contrasting effects in different types of hematopoietic malignancies. 

*Tet3* KO mice did not display any significant hematopoietic abnormalities [92], and a recent study showed that aged *Tet3* KO mice harboring haploinsufficiency of the *Tet2* allele ultimately developed AML after a long latency [108]. Interestingly, in the leukemic mice, the remaining *Tet2* allele was lost during the development of AML. These results suggest the tumor suppressor function of TET3 in malignant hematopoiesis. In contrast, TET3 expression was shown to be aberrant in AML patients, with its depletion suppressing the growth of AML cells in vitro and in vivo. The enforced expression of TET3 substantially impaired myeloid, but not erythroid, colony formation, suggesting its oncogenic roles. Further studies are required to precisely define the role of TET3 in oncogenesis.

### 5.3. TET2 in HSC Self-Renewal and Lineage Commitment

Despite the antagonistic biochemical activities, with DNMT3A yielding the 5mC mark and TET2 erasing it, deletion of *Tet2* in mice paradoxically leads to similar phenotypic outcomes as a DNMT3A LOF in terms of enhanced HSC self-renewal, clonal hematopoiesis, impeded differentiation (on a per-HSC basis), and oncogenesis [43,81,94,109] (Table 2). Although there are subtle differences in the degree of their impacts when compared in parallel, the overall direction of the phenotypic changes is the same in both murine models. Transplantation of *Tet2* KO bone marrow cells or *Dnmt3a* KO HSCs showed augmented peripheral blood chimerism in a cell-intrinsic manner [77,95,110,111,112]. The loss of TET2 and DNMT3A influenced HSC self-renewal to a different extent: in serial transplantation assays, the ability of TET2 KO HSCs to self-renew was transiently increased during early passages of transplantation but decreased up to the level in WT HSCs after the third transplantation, although DNMT3A KO HSCs could regenerate almost indefinitely in vivo [81,82]. Furthermore, while DNMT3A loss more specifically affected HSCs [77,80,81,82], TET2 deficiency exhibited a broader effect on HSPCs. Indeed, the primary impact of the TET2 LOF seems to be driving skewed myeloid differentiation of committed progenitors rather than long-term HSCs [81], in agreement with frequent occurrences of *TET2* mutations in myeloid-primed progenitors in CHIP [74]. 

In contrast, a recent single-cell RNA-sequencing analysis highlights that the opposing effects of DNMT3A and TET2 loss on the DNA methylation status indeed have antagonistic effects on the early HSPC lineage specification [113]. TET2 loss in HSCs favored differentiation skews toward myelomonocytic over erythroid progenitors, while DNMT3A loss caused an opposite shift. Mechanistically, this disturbed hematopoietic lineage commitment was attributed to opposing biases in transcriptional priming, with TET2 and DNMT3A LOF favoring the myelomonocytic and erythroid lineages, respectively, in uncommitted HSCs. Consistent with the notion that direct inhibition of TF binding is considered the primary mode of gene silencing by DNA methylation [114,115], the chromatin accessibility of key lineage-determining TFs was particularly susceptible to methylation changes, and strikingly, its sensitivity was determined by the CpG density of the binding motifs [113]. As the TF binding motif had a higher CpG enrichment, it was more readily inactivated by hypermethylation. Interestingly, the DNA-binding motifs of erythroid TFs had a higher CpG content than those of myelomonocytic TFs. Thus, erythroid TFs (e.g., TAL1 and KLF1) were strongly inactivated by TET2 loss-induced hypermethylation, with an opposite effect being observed in DNMT3A loss-induced hypomethylation. However, myelomonocytic TFs (e.g., IRF8 and SP1) were not significantly affected due to their low CpG content in their binding sites. As a result, TET2 loss caused myelomonocytic skews in HSC priming, whereas DNMT3A loss induced erythroid skews. Thus, DNMT3A and TET2 exert antagonistic effects on genome-wide methylation in HSCs, which is connected to differentiation skews through the differences in CpG enrichment of the TF binding site. 

**Table 2:** Hematopoietic phenotypes of TET-deficient animal models.

Genotype	Major KO Mice Phenotype	Hematologic Malignancy	References
*Tet1^−/−^*	Increased HSC self-renewal; skewed differentiation toward B lineage; enhanced colony formation in vitro; accumulation of DNA damage	B-cell lymphoma (median survival, 22 mo)	[102]
*Tet1^−/−^*; bone marrow transfer after retroviral expression of shTet1	TET1 induction by MLL fusions	Delayed MLL-AF9-induced leukemogenesis	[103]
*Tet3^fl/fl^ Vav-Cre*	Normal tri-lineage differentiation; augmented repopulation capacity	Not observed	[92]
*Tet2^fl/+^ Mx-Cre; Tet3^fl/+^ Mx-Cre*; *Tet2^fl/fl^ Tet3^fl/fl^ Mx-Cre*	Inactivation of nontargeted *Tet2* or *Tet3* allele in AMLs in the single KO mice	AML in *Tet2/3* double KO (median survival, ~10.7 wk); AML with a longer latencies in *Tet2* or *Tet3* single KO (median survival, ~27 wk)	[108]
*Tet2^fl/fl^ Mx-Cre* or *Vav-Cre*	Limited HSC self-renewal in serial transplantation; profound myeloid skewing	Myeloid malignancy (MPD); accelerated Flt3^ITD^-driven AML development	[81]
*Tet2* gene trap	Enhanced self-renewal and long-term repopulating capacity of fetal liver HSCs; myeloid skewing	Not observed	[109]
*Tet2* gene trap; *Tet2^fl/fl^ Mx1-Cre*	Expansion of HSPC and myeloid progenitors; competitive repopulation advantage; myeloid expansion	CMML-like disease (gene trap)	[95]
*Tet2^−/−^*	Expansion of HSPC and myeloid progenitors; competitive repopulation advantage; skewed differentiation toward myeloid lineage in vitro	Not observed	[110]
*Tet2* gene trap	Expansion of HSPC and myeloid progenitors; competitive repopulation advantage; profound leukocytosis	Myeloid malignancy (~30% of KO mice; CMML, MPN, MDS, etc.)	[111]
*Tet2^fl/fl^ Vav-Cre*	Expansion of HSPC and myeloid progenitors; competitive repopulation advantage	CMML-like disease	[112]
*Tet2* gene trap (transplantation of fetal liver cells)	Anemia, lymphopenia, thrombocytopenia, dysplasia of myeloid cells	MDS- or CMML-like diseases	[116]
*Tet2^fl/fl^ Vav1-Cre* or *LysM-Cre*	Suppression of leukemogenesis by WT, but not catalytically inactive TET2 mutant	CMML (50%) or MPD (33.3%) in *Tet2^fl/fl^ Vav1-Cre*; no malignancy in *Tet2^fl/fl^ LysM-Cre*	[117]
*Tet2^fl/fl^ Tet3^fl/fl^ Mx1-Cre* or *CreERT2*	Rapid myeloid expansion; strong myeloid skewing; fully-penetrant, transplantable, lethal myeloid leukemia within 3–7 wk	Myeloid leukemia (100%, transplantable, median survival, 1 mo)	[118]
*Tet2^−/−^*, *Tet2^mut/mut^*	Distinct gene expression profiles in both models	Myeloid (44.4%) and lymphoid (38.9%) diseases in *Tet2^−/−^* mice; myeloid malignancy (78.5%) in *Tet2^mutmut^* mice	[119]
*Tet2* gene trap	Outgrowth of Tfh-like cells in the spleen; lymphomas with similar gene expression patterns as Tfh cells; aberrant DNA methylation and hydroxymethylation	T-cell lymphoma with Tfh features (median survival, ~67 wk)	[120]
*Tet2^fl/fl^ Vav-Cre* or *CD19-Cre*	Defective class switch recombination and affinity maturation; germinal center hyperplasia; impaired plasma cell differentiation; mimics CREBBP mutant	Not observed	[121]
*Tet2^fl/fl^ Vav-Cre*	Hypermethylation in germinal center B cells; impaired B-cell TF by loss of enhancer 5hmC	Not observed	[122]
*Tet2^fl/fl^ CD19-Cre*	B-cell accumulation; abnormalities in the B1-cell subset; acquisition of AID-mediated mutations in *Tet2* KO tumors	B-cell malignancy (50% of mice)	[123]
*Tet1^−/−^ Tet2^−/−^*	Increased common lymphoid progenitor and B-cell colony formation; increased short-term, but not long-term, repopulation capacity	B-cell malignancy (median survival, 20 mo, transplantable)	[124]
*Tet2^fl/fl^ Tet3^fl/fl^ CD19-Cre*	Increased G-quadruplexes and R-loops; increased DNA double-strand breaks at immunoglobulin switch regions	B-cell lymphoma (median survival, 20 wk; DLBLC-like);	[125]
*Tet2^fl/fl^ Tet3^fl/fl^ CreERT2*	Impaired class switch recombination via impaired AID expression; impaired 5hmC modification and chromatin accessibility of super-enhancers in the *Aicda* locus	Not observed	[56]

### 5.4. Dysregulation of TET2 in Hematologic Malignancies

Although *TET2* mutations are prevalent in hematologic neoplasms, *TET2* mutation alone is insufficient to potently drive hematopoietic transformation (Table 2). Indeed, only a subset of TET2 KO mice developed myeloid and/or lymphoid malignancies with partial penetrance and very long latencies (~2 years) [92,93,94]. Myeloproliferation and lethal neoplasia resembling human CMML, MPN, AML, and MDS were most prominent in mice when *Tet2* was deleted in all hematopoietic cells, including HSCs [95,108,111,112,116]. However, with the deletion of *Tet2* in differentiated myeloid cells (using LysM-Cre), no malignancies were observed, indicating that the TET2 LOF needs to occur in early HSPCs to initiate hematologic diseases [117]. Myeloid leukemogenesis was strikingly potentiated in mice doubly deficient for TET2 and TET3, resulting in highly aggressive, fully-penetrant, and transplantable myeloid leukemias within three to seven weeks [118]. Furthermore, the catalytic activity of TET2 was initially shown to be essential for the suppression of leukemogenesis [117], but a later study showed that both TET2 KO and catalytic mutant mice developed malignancies with distinct disease spectra: while TET2 KO mice developed both myeloid and lymphoid malignancies, the catalytic mutant mice almost exclusively developed myeloid malignancies [119]. Interestingly, the *Tet3* allele was lost during leukemic progression to AML in the TET2 catalytic mutant mice, suggesting that the TET2 catalytic activity might be important for genome stability.

TET2 deficiency also drives lymphomagenesis (Table 2). TET2 depletion in gene trap mice led to T-cell lymphomas with Tfh features after a long latency (median ~67 weeks) [120]. Consistent with frequent *TET2* mutations in DLBCL, *Tet2* deletion in HSPCs or B cells in mice (using Vav-Cre or CD19-Cre) caused germinal center hyperplasia and impaired plasma cell differentiation by impairing germinal center B cell epigenome and transcriptome [121,122], ultimately developing B-cell lymphoma [123]. Furthermore, mice with combined deletion of *Tet1* and *Tet2* in HSPCs (using Mx-Cre) developed lethal B cell malignancies and died within 20 months [124]. Furthermore, mice with a combined deletion of Tet2 and Tet3 in B cells (using CD19-Cre) rapidly developed DLBCL-like tumors from germinal center B cells with complete penetrance and a median survival of ~20 weeks [125]. Notably, the expanded cells in these mice robustly accumulated DNA damage associated with increased G-quadruplex and R-loop structures [125].

Intriguingly, preleukemic hematopoietic cells from *Csf3r/RUNX1* mutant mice progressed to AML by acquiring *CXXC4^ITD^* (ITD, internal tandem duplication) mutation as a second hit [126]. The *CXXC4* (also called *IDAX*) gene was originally part of an ancestral *TET2* gene. During evolution, it underwent chromosomal rearrangement and was separated from the *TET2* gene, forming a separate gene that encodes the CXXC domain of the ancestral TET2 protein (Figure 1a) [127]. The accumulated *CXXC4^ITD^* mutations elevated levels of IDAX proteins by increasing their stability. Consistent with the antagonistic effect of IDAX on TET2 protein levels, as previously reported [127], *CXXC4^ITD^* mutations decreased TET2 protein levels, which seemed to drive the malignant transformation to AML [126]. 

### 5.5. Cooperation with Additional Mutations

As described previously, *TET2* mutations seem to increase the pool of pre-leukemic HSPCs that are susceptible to subsequent mutations (i.e., second hits) to develop into full-blown diseases (Figure 2). Indeed, *TET2*-mutated cancers often harbor various cooperating mutations in genes encoding FLT3, ASXL1, JAK2, EZH2, NRAS, KIT, RHOA, DNMT3A, SRSF2, AML-ETO1, etc. [92,93,128,129]. The outcome of this cooperation has been functionally evaluated in TET2 KO mice harboring one of these mutations, such as *Flt3^ITD^* [130], *Asxl1* [131], *JAK2^V617F^* [132], *Ezh2* [133], *Nras* [134], *KIT^D816V^* [135], *RhoA^G17V^* [136], *DNMT3A^R882H^* [137], *SRSF2^P95H^* [138], *AML-ETO* [139], etc. Overall, when combined with a TET2 LOF, these mutations substantially accelerated the development of various types of hematologic neoplasms with significantly shortened latencies; major phenotypes of the mice are summarized in [93]. 

It is notable that despite the opposing effects of DNMT3A and TET2 loss in shaping early myeloid versus lymphoid biases of early progenitors, loss of either protein in mice leads to similar long-term outcomes in terms of development of lethal malignancies [81,108]. However, TET2- and DNMT3A KO mice responded differently to even the same cooperating mutations: when combined with *Flt3^ITD^* mutation, TET2 KO mice died more rapidly of mostly MPNs, while DNMT3A KO mice survived longer but eventually developed mixed phenotype acute leukemia [81]. Despite the epistatic relationship in the DNA methylation-hydroxymethylation pathway, *DNMT3A* and *TET2* mutations are often concurrent in lymphoma and leukemia patients [97,98,99,100], suggesting that both enzymes sometimes work in parallel to produce a common result. Consistent with this, the combined deletion of *Dnmt3a* and *Tet2* in mice displayed synergism to enhance the competitive advantage and expression of lineage-specific TFs in HSCs, eventually resulting in an accelerated progression of multiple malignancies, including CMML-like diseases and B/T-cell lymphoma [140]. A similar synergistic impact was also observed in TET2 KO mice expressing *DNMT3A^R882H^* mutation [137]. However, it remains poorly understood why the LOF of DNMT3A and TET2 results in divergent effects in early HSPC commitment but more convergent effects later.

## 6. TET Modulation of Inflammation in Clonal Hematopoiesis

Chronic low-grade inflammation is a hallmark of aging and thus has gained considerable attention from hematologists due to its potential role as a cell-extrinsic contributor to CHIP (Figure 2). Bone marrow niches play an active role in the initiation and progression of hematologic cancers, particularly myeloid malignancies [141]. Niche-driven pro-inflammatory signals such as tumor necrosis factor α, interleukin-6 (IL-6), and IL-1 create a hostile environment for normal HSCs and can contribute to malignancy by conferring a competitive advantage on HSCs harboring specific mutations [141]. Previous studies have identified inflammation as a key determinant for the selective advantage of TET2 KO HSPC and disease progression. Notably, TET2 KO HSPC resisted inflammatory signals. Upon pro-inflammatory stimuli such as lipopolysaccharide or diabetes-induced hyperglycemia, TET2 KO HSPC and mature myeloid cells were amplified, increasing systemic levels of IL-6 [142,143]. Then, IL-6 led to hyperactivation of the Shp2/Stat3/Morrbid pathway in TET2 KO HSPCs. Given that Morrbid is an anti-apoptotic long noncoding RNA that selectively suppresses pro-apoptotic *Bim* expression, TET2 loss may provide preleukemic HSPCs with a survival advantage to drive clonal expansion in an inflammatory milieu [142,143]. Likewise, TET2 loss in murine and human HSPCs also augmented clonal advantage under an inflammatory environment containing TNF-α [144]. Another study showed that TET2 deficiency led to systemic bacterial dissemination and elevated IL-6 production by disrupting the integrity of the intestinal barrier, which was critical for preleukemic myeloproliferation. Notably, the TET2 loss-induced myeloproliferation could be substantially reversed using antibiotic treatment or under germ-free conditions [145]. In agreement with this observation, the suppression of gut microbiota-dependent inflammation with antibiotics or pharmacological inhibition of TNF-α also suppressed the expansion of myeloid and lymphoid malignancies in vivo [146]. Together, these results indicate that inflammatory signals confer on TET2-mutant HSPC a competitive advantage to drive clonal expansion. Intriguingly, TET2 acted as a cell-intrinsic suppressor of the inflammatory response in myeloid cells by recruiting HDAC2 to suppress IL-6 [147,148]. Thus, CHIP-associated *TET2* mutations may establish a positive feedback loop: the TET2-mutant myeloid cells potentiate CHIP by amplifying the inflammatory environment through elevated IL-6 secretion, which then further augments the competitive advantage and amplification of TET2-mutant HSPCs in the bone marrow by exerting a negative effect on the normal counterparts. 

## 7. Perspectives and Conclusions

Recent systematic studies involving the next-generation sequencing of tumor genomes have shown that mutations in genes encoding two key DNA-modifying enzymes, DNMT3A and TET2, are recurrent events in CHIP as well as in a wide range of hematologic malignancies. These mutations occur at an early HSPC stage, and then the expanded premalignant clones acquire additional mutations to further develop into frank malignancies (Figure 2). Thus, the advancement of next-generation sequencing may enable the early detection of hematologic neoplasms. However, only a subset of aged individuals with CHIP develops full-blown malignancy, so factors determining the progression of CHIP clones to malignancy remain to be fully determined.

**Figure 3:**
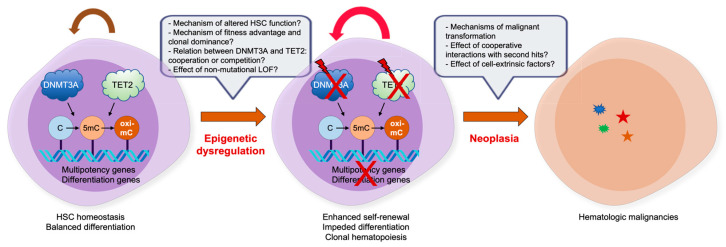
Impact of DNMT and TET mutations in CHIP and hematologic malignancies. Despite the progress we have made in the last decades to decipher the role of epimutations, such as DNMT3A and TET2 LOF mutations, there are still many outstanding issues. For details, refer to the text.

The mutational profiling in patient samples and analyses of various murine models mimicking LOF mutations in *DNMT3A* and *TET2*, alone or in combination, have made significant progress in our understanding of the underlying molecular mechanisms through which these epigenetic regulators influence normal and malignant hematopoiesis. Nonetheless, there are several key areas in which many unresolved questions remain (Figure 3). Foremost among these would be why *DNMT3A* and *TET2* mutations have profound effects on HSC clonal expansion and progression to malignancies, how they display overlapping and nonoverlapping effects, how they interact with a variety of second hits during clonal evolution to full-blown malignancies, how they work together with cell-extrinsic factors to establish clonal dominance, and whether the LOF in the absence of coding region mutations also leads to similar effects on CHIP and oncogenesis. It will also be interesting to define whether the combined LOF of TET proteins, with TET2 and TET1/3 being inactivated genetically and epigenetically, respectively, display more robust driving effects in CHIP and oncogenesis. Fundamentally, both proteins are crucial regulators of gene expression that control DNA methylation status, TF binding, DNA flexibility and integrity, chromatin architecture and stability, histone modifications, and even three-dimensional genome interactions. Any aberrations occurring in these processes may have critical impacts on the establishment of clonal hematopoiesis and subsequent tumor progression. Therefore, further characterization of the epigenetic regulation of clonal hematopoiesis and malignant transformation is warranted to decipher the precise transformation mechanism.

## Data Availability

Not applicable.

## References

[1] Jones P.A. (2012). Functions of DNA methylation: Islands, start sites, gene bodies and beyond. Nat. Rev. Genet..

[2] Dan J., Chen T. (2022). Genetic Studies on Mammalian DNA Methyltransferases. Adv. Exp. Med. Biol..

[3] Greenberg M.V.C., Bourc’his D. (2019). The diverse roles of DNA methylation in mammalian development and disease. Nat. Rev. Mol. Cell Biol..

[4] Bock C. (2012). Analysing and interpreting DNA methylation data. Nat. Rev. Genet..

[5] Bestor T.H., Bourc’his D. (2004). Transposon silencing and imprint establishment in mammalian germ cells. Cold Spring Harb. Symp. Quant. Biol..

[6] Jansz N. (2019). DNA methylation dynamics at transposable elements in mammals. Essays Biochem..

[7] Xie M., Hong C., Zhang B., Lowdon R.F., Xing X., Li D., Zhou X., Lee H.J., Maire C.L., Ligon K.L. (2013). DNA hypomethylation within specific transposable element families associates with tissue-specific enhancer landscape. Nat. Genet..

[8] Laurent L., Wong E., Li G., Huynh T., Tsirigos A., Ong C.T., Low H.M., Kin Sung K.W., Rigoutsos I., Loring J. (2010). Dynamic changes in the human methylome during differentiation. Genome Res..

[9] Yang X., Han H., De Carvalho D.D., Lay F.D., Jones P.A., Liang G. (2014). Gene body methylation can alter gene expression and is a therapeutic target in cancer. Cancer Cell.

[10] Taryma-Lesniak O., Sokolowska K.E., Wojdacz T.K. (2021). Short history of 5-methylcytosine: From discovery to clinical applications. J. Clin. Pathol..

[11] Cancer Genome Atlas Research Network (2008). Comprehensive genomic characterization defines human glioblastoma genes and core pathways. Nature.

[12] Cancer Genome Atlas Research Network (2011). Integrated genomic analyses of ovarian carcinoma. Nature.

[13] Cancer Genome Atlas Research Network (2012). Comprehensive molecular characterization of human colon and rectal cancer. Nature.

[14] Stransky N., Egloff A.M., Tward A.D., Kostic A.D., Cibulskis K., Sivachenko A., Kryukov G.V., Lawrence M.S., Sougnez C., McKenna A. (2011). The mutational landscape of head and neck squamous cell carcinoma. Science.

[15] Cedar H., Bergman Y. (2012). Programming of DNA methylation patterns. Annu. Rev. Biochem..

[16] Schubeler D. (2015). Function and information content of DNA methylation. Nature.

[17] Nabel C.S., Manning S.A., Kohli R.M. (2012). The curious chemical biology of cytosine: Deamination, methylation, and oxidation as modulators of genomic potential. ACS Chem. Biol..

[18] Du J., Johnson L.M., Jacobsen S.E., Patel D.J. (2015). DNA methylation pathways and their crosstalk with histone methylation. Nat. Rev. Mol. Cell Biol..

[19] Lyko F. (2018). The DNA methyltransferase family: A versatile toolkit for epigenetic regulation. Nat. Rev. Genet..

[20] Ooi S.K., O’Donnell A.H., Bestor T.H. (2009). Mammalian cytosine methylation at a glance. J. Cell Sci..

[21] Lister R., Pelizzola M., Dowen R.H., Hawkins R.D., Hon G., Tonti-Filippini J., Nery J.R., Lee L., Ye Z., Ngo Q.M. (2009). Human DNA methylomes at base resolution show widespread epigenomic differences. Nature.

[22] Stadler M.B., Murr R., Burger L., Ivanek R., Lienert F., Scholer A., van Nimwegen E., Wirbelauer C., Oakeley E.J., Gaidatzis D. (2011). DNA-binding factors shape the mouse methylome at distal regulatory regions. Nature.

[23] Ziller M.J., Gu H., Muller F., Donaghey J., Tsai L.T., Kohlbacher O., De Jager P.L., Rosen E.D., Bennett D.A., Bernstein B.E. (2013). Charting a dynamic DNA methylation landscape of the human genome. Nature.

[24] Chuang L.S., Ian H.I., Koh T.W., Ng H.H., Xu G., Li B.F. (1997). Human DNA-(cytosine-5) methyltransferase-PCNA complex as a target for p21WAF1. Science.

[25] Bostick M., Kim J.K., Esteve P.O., Clark A., Pradhan S., Jacobsen S.E. (2007). UHRF1 plays a role in maintaining DNA methylation in mammalian cells. Science.

[26] Sharif J., Muto M., Takebayashi S., Suetake I., Iwamatsu A., Endo T.A., Shinga J., Mizutani-Koseki Y., Toyoda T., Okamura K. (2007). The SRA protein Np95 mediates epigenetic inheritance by recruiting Dnmt1 to methylated DNA. Nature.

[27] Arita K., Ariyoshi M., Tochio H., Nakamura Y., Shirakawa M. (2008). Recognition of hemi-methylated DNA by the SRA protein UHRF1 by a base-flipping mechanism. Nature.

[28] Avvakumov G.V., Walker J.R., Xue S., Li Y., Duan S., Bronner C., Arrowsmith C.H., Dhe-Paganon S. (2008). Structural basis for recognition of hemi-methylated DNA by the SRA domain of human UHRF1. Nature.

[29] Xu C., Corces V.G. (2018). Nascent DNA methylome mapping reveals inheritance of hemimethylation at CTCF/cohesin sites. Science.

[30] Vertino P.M., Yen R.W., Gao J., Baylin S.B. (1996). De novo methylation of CpG island sequences in human fibroblasts overexpressing DNA (cytosine-5-)-methyltransferase. Mol. Cell. Biol..

[31] Haggerty C., Kretzmer H., Riemenschneider C., Kumar A.S., Mattei A.L., Bailly N., Gottfreund J., Giesselmann P., Weigert R., Brandl B. (2021). Dnmt1 has de novo activity targeted to transposable elements. Nat. Struct. Mol. Biol..

[32] Chen T., Ueda Y., Dodge J.E., Wang Z., Li E. (2003). Establishment and maintenance of genomic methylation patterns in mouse embryonic stem cells by Dnmt3a and Dnmt3b. Mol. Cell. Biol..

[33] Liu Y., Xu Z., Shi J., Zhang Y., Yang S., Chen Q., Song C., Geng S., Li Q., Li J. (2022). DNA methyltransferases are complementary in maintaining DNA methylation in embryonic stem cells. iScience.

[34] Okano M., Bell D.W., Haber D.A., Li E. (1999). DNA methyltransferases Dnmt3a and Dnmt3b are essential for de novo methylation and mammalian development. Cell.

[35] Hsieh C.L. (1999). In vivo activity of murine de novo methyltransferases, Dnmt3a and Dnmt3b. Mol. Cell. Biol..

[36] Feng J., Zhou Y., Campbell S.L., Le T., Li E., Sweatt J.D., Silva A.J., Fan G. (2010). Dnmt1 and Dnmt3a maintain DNA methylation and regulate synaptic function in adult forebrain neurons. Nat. Neurosci..

[37] Jeong M., Sun D., Luo M., Huang Y., Challen G.A., Rodriguez B., Zhang X., Chavez L., Wang H., Hannah R. (2014). Large conserved domains of low DNA methylation maintained by Dnmt3a. Nat. Genet..

[38] Liang G., Chan M.F., Tomigahara Y., Tsai Y.C., Gonzales F.A., Li E., Laird P.W., Jones P.A. (2002). Cooperativity between DNA methyltransferases in the maintenance methylation of repetitive elements. Mol. Cell. Biol..

[39] Jones P.A., Liang G. (2009). Rethinking how DNA methylation patterns are maintained. Nat. Rev. Genet..

[40] Hackett J.A., Surani M.A. (2013). DNA methylation dynamics during the mammalian life cycle. Philos. Trans. R. Soc. Lond. B Biol. Sci..

[41] Hackett J.A., Sengupta R., Zylicz J.J., Murakami K., Lee C., Down T.A., Surani M.A. (2013). Germline DNA demethylation dynamics and imprint erasure through 5-hydroxymethylcytosine. Science.

[42] Tahiliani M., Koh K.P., Shen Y., Pastor W.A., Bandukwala H., Brudno Y., Agarwal S., Iyer L.M., Liu D.R., Aravind L. (2009). Conversion of 5-methylcytosine to 5-hydroxymethylcytosine in mammalian DNA by MLL partner TET1. Science.

[43] Ko M., Huang Y., Jankowska A.M., Pape U.J., Tahiliani M., Bandukwala H.S., An J., Lamperti E.D., Koh K.P., Ganetzky R. (2010). Impaired hydroxylation of 5-methylcytosine in myeloid cancers with mutant TET2. Nature.

[44] He Y.F., Li B.Z., Li Z., Liu P., Wang Y., Tang Q., Ding J., Jia Y., Chen Z., Li L. (2011). Tet-mediated formation of 5-carboxylcytosine and its excision by TDG in mammalian DNA. Science.

[45] Ito S., Shen L., Dai Q., Wu S.C., Collins L.B., Swenberg J.A., He C., Zhang Y. (2011). Tet proteins can convert 5-methylcytosine to 5-formylcytosine and 5-carboxylcytosine. Science.

[46] Yu Z., Genest P.A., ter Riet B., Sweeney K., DiPaolo C., Kieft R., Christodoulou E., Perrakis A., Simmons J.M., Hausinger R.P. (2007). The protein that binds to DNA base J in trypanosomatids has features of a thymidine hydroxylase. Nucleic Acids Res..

[47] Iyer L.M., Abhiman S., Aravind L. (2011). Natural history of eukaryotic DNA methylation systems. Prog. Mol. Biol. Transl. Sci..

[48] Iyer L.M., Tahiliani M., Rao A., Aravind L. (2009). Prediction of novel families of enzymes involved in oxidative and other complex modifications of bases in nucleic acids. Cell Cycle.

[49] Shen L., Song C.X., He C., Zhang Y. (2014). Mechanism and function of oxidative reversal of DNA and RNA methylation. Annu. Rev. Biochem..

[50] Crawford D.J., Liu M.Y., Nabel C.S., Cao X.J., Garcia B.A., Kohli R.M. (2016). Tet2 Catalyzes Stepwise 5-Methylcytosine Oxidation by an Iterative and de novo Mechanism. J. Am. Chem. Soc..

[51] Pastor W.A., Aravind L., Rao A. (2013). TETonic shift: Biological roles of TET proteins in DNA demethylation and transcription. Nat. Rev. Mol. Cell Biol..

[52] Wu X., Zhang Y. (2017). TET-mediated active DNA demethylation: Mechanism, function and beyond. Nat. Rev. Genet..

[53] Hashimoto H., Liu Y., Upadhyay A.K., Chang Y., Howerton S.B., Vertino P.M., Zhang X., Cheng X. (2012). Recognition and potential mechanisms for replication and erasure of cytosine hydroxymethylation. Nucleic Acids Res..

[54] Otani J., Kimura H., Sharif J., Endo T.A., Mishima Y., Kawakami T., Koseki H., Shirakawa M., Suetake I., Tajima S. (2013). Cell cycle-dependent turnover of 5-hydroxymethyl cytosine in mouse embryonic stem cells. PLoS ONE.

[55] Inoue A., Zhang Y. (2011). Replication-dependent loss of 5-hydroxymethylcytosine in mouse preimplantation embryos. Science.

[56] Lio C.J., Shukla V., Samaniego-Castruita D., Gonzalez-Avalos E., Chakraborty A., Yue X., Schatz D.G., Ay F., Rao A. (2019). TET enzymes augment activation-induced deaminase (AID) expression via 5-hydroxymethylcytosine modifications at the Aicda superenhancer. Sci. Immunol..

[57] Lio C.J., Yue X., Lopez-Moyado I.F., Tahiliani M., Aravind L., Rao A. (2020). TET methylcytosine oxidases: New insights from a decade of research. J. Biosci..

[58] Schomacher L., Niehrs C. (2017). DNA repair and erasure of 5-methylcytosine in vertebrates. BioEssays.

[59] Maiti A., Drohat A.C. (2011). Thymine DNA glycosylase can rapidly excise 5-formylcytosine and 5-carboxylcytosine: Potential implications for active demethylation of CpG sites. J. Biol. Chem..

[60] Zhang L., Lu X., Lu J., Liang H., Dai Q., Xu G.L., Luo C., Jiang H., He C. (2012). Thymine DNA glycosylase specifically recognizes 5-carboxylcytosine-modified DNA. Nat. Chem. Biol..

[61] Wang D., Wu W., Callen E., Pavani R., Zolnerowich N., Kodali S., Zong D., Wong N., Noriega S., Nathan W.J. (2022). Active DNA demethylation promotes cell fate specification and the DNA damage response. Science.

[62] Liu M.Y., Torabifard H., Crawford D.J., DeNizio J.E., Cao X.J., Garcia B.A., Cisneros G.A., Kohli R.M. (2017). Mutations along a TET2 active site scaffold stall oxidation at 5-hydroxymethylcytosine. Nat. Chem. Biol..

[63] Morgan H.D., Dean W., Coker H.A., Reik W., Petersen-Mahrt S.K. (2004). Activation-induced cytidine deaminase deaminates 5-methylcytosine in DNA and is expressed in pluripotent tissues: Implications for epigenetic reprogramming. J. Biol. Chem..

[64] Guo J.U., Su Y., Zhong C., Ming G.L., Song H. (2011). Hydroxylation of 5-methylcytosine by TET1 promotes active DNA demethylation in the adult brain. Cell.

[65] Cortellino S., Xu J., Sannai M., Moore R., Caretti E., Cigliano A., Le Coz M., Devarajan K., Wessels A., Soprano D. (2011). Thymine DNA glycosylase is essential for active DNA demethylation by linked deamination-base excision repair. Cell.

[66] Guo F., Li X., Liang D., Li T., Zhu P., Guo H., Wu X., Wen L., Gu T.P., Hu B. (2014). Active and passive demethylation of male and female pronuclear DNA in the mammalian zygote. Cell Stem Cell.

[67] Schiesser S., Hackner B., Pfaffeneder T., Muller M., Hagemeier C., Truss M., Carell T. (2012). Mechanism and stem-cell activity of 5-carboxycytosine decarboxylation determined by isotope tracing. Angew. Chem. Int. Ed..

[68] Ferrando A.A., Lopez-Otin C. (2017). Clonal evolution in leukemia. Nat. Med..

[69] Florez M.A., Tran B.T., Wathan T.K., DeGregori J., Pietras E.M., King K.Y. (2022). Clonal hematopoiesis: Mutation-specific adaptation to environmental change. Cell Stem Cell.

[70] Challen G.A., Goodell M.A. (2020). Clonal hematopoiesis: Mechanisms driving dominance of stem cell clones. Blood.

[71] Fabre M.A., de Almeida J.G., Fiorillo E., Mitchell E., Damaskou A., Rak J., Orru V., Marongiu M., Chapman M.S., Vijayabaskar M.S. (2022). The longitudinal dynamics and natural history of clonal haematopoiesis. Nature.

[72] Abelson S., Collord G., Ng S.W.K., Weissbrod O., Mendelson Cohen N., Niemeyer E., Barda N., Zuzarte P.C., Heisler L., Sundaravadanam Y. (2018). Prediction of acute myeloid leukaemia risk in healthy individuals. Nature.

[73] Shlush L.I., Zandi S., Mitchell A., Chen W.C., Brandwein J.M., Gupta V., Kennedy J.A., Schimmer A.D., Schuh A.C., Yee K.W. (2014). Identification of pre-leukaemic haematopoietic stem cells in acute leukaemia. Nature.

[74] Buscarlet M., Provost S., Zada Y.F., Bourgoin V., Mollica L., Dube M.P., Busque L. (2018). Lineage restriction analyses in CHIP indicate myeloid bias for TET2 and multipotent stem cell origin for DNMT3A. Blood.

[75] Rossi L., Lin K.K., Boles N.C., Yang L., King K.Y., Jeong M., Mayle A., Goodell M.A. (2012). Less is more: Unveiling the functional core of hematopoietic stem cells through knockout mice. Cell Stem Cell.

[76] Broske A.M., Vockentanz L., Kharazi S., Huska M.R., Mancini E., Scheller M., Kuhl C., Enns A., Prinz M., Jaenisch R. (2009). DNA methylation protects hematopoietic stem cell multipotency from myeloerythroid restriction. Nat. Genet..

[77] Challen G.A., Sun D., Jeong M., Luo M., Jelinek J., Berg J.S., Bock C., Vasanthakumar A., Gu H., Xi Y. (2012). Dnmt3a is essential for hematopoietic stem cell differentiation. Nat. Genet..

[78] Tadokoro Y., Ema H., Okano M., Li E., Nakauchi H. (2007). De novo DNA methyltransferase is essential for self-renewal, but not for differentiation, in hematopoietic stem cells. J. Exp. Med..

[79] Trowbridge J.J., Snow J.W., Kim J., Orkin S.H. (2009). DNA methyltransferase 1 is essential for and uniquely regulates hematopoietic stem and progenitor cells. Cell Stem Cell.

[80] Challen G.A., Sun D., Mayle A., Jeong M., Luo M., Rodriguez B., Mallaney C., Celik H., Yang L., Xia Z. (2014). Dnmt3a and Dnmt3b have overlapping and distinct functions in hematopoietic stem cells. Cell Stem Cell.

[81] Ostrander E.L., Kramer A.C., Mallaney C., Celik H., Koh W.K., Fairchild J., Haussler E., Zhang C.R.C., Challen G.A. (2020). Divergent Effects of Dnmt3a and Tet2 Mutations on Hematopoietic Progenitor Cell Fitness. Stem Cell Rep..

[82] Jeong M., Park H.J., Celik H., Ostrander E.L., Reyes J.M., Guzman A., Rodriguez B., Lei Y., Lee Y., Ding L. (2018). Loss of Dnmt3a Immortalizes Hematopoietic Stem Cells In Vivo. Cell Rep..

[83] Yang L., Rau R., Goodell M.A. (2015). DNMT3A in haematological malignancies. Nat. Rev. Cancer.

[84] Russler-Germain D.A., Spencer D.H., Young M.A., Lamprecht T.L., Miller C.A., Fulton R., Meyer M.R., Erdmann-Gilmore P., Townsend R.R., Wilson R.K. (2014). The R882H DNMT3A mutation associated with AML dominantly inhibits wild-type DNMT3A by blocking its ability to form active tetramers. Cancer Cell.

[85] Cole C.B., Russler-Germain D.A., Ketkar S., Verdoni A.M., Smith A.M., Bangert C.V., Helton N.M., Guo M., Klco J.M., O’Laughlin S. (2017). Haploinsufficiency for DNA methyltransferase 3A predisposes hematopoietic cells to myeloid malignancies. J. Clin. Investig..

[86] Peters S.L., Hlady R.A., Opavska J., Klinkebiel D., Pirruccello S.J., Talmon G.A., Sharp J.G., Wu L., Jaenisch R., Simpson M.A. (2014). Tumor suppressor functions of Dnmt3a and Dnmt3b in the prevention of malignant mouse lymphopoiesis. Leukemia.

[87] Mayle A., Yang L., Rodriguez B., Zhou T., Chang E., Curry C.V., Challen G.A., Li W., Wheeler D., Rebel V.I. (2015). Dnmt3a loss predisposes murine hematopoietic stem cells to malignant transformation. Blood.

[88] Celik H., Mallaney C., Kothari A., Ostrander E.L., Eultgen E., Martens A., Miller C.A., Hundal J., Klco J.M., Challen G.A. (2015). Enforced differentiation of Dnmt3a-null bone marrow leads to failure with c-Kit mutations driving leukemic transformation. Blood.

[89] Guryanova O.A., Lieu Y.K., Garrett-Bakelman F.E., Spitzer B., Glass J.L., Shank K., Martinez A.B., Rivera S.A., Durham B.H., Rapaport F. (2016). Dnmt3a regulates myeloproliferation and liver-specific expansion of hematopoietic stem and progenitor cells. Leukemia.

[90] Schulze I., Rohde C., Scheller-Wendorff M., Baumer N., Krause A., Herbst F., Riemke P., Hebestreit K., Tschanter P., Lin Q. (2016). Increased DNA methylation of Dnmt3b targets impairs leukemogenesis. Blood.

[91] Xu J., Wang Y.Y., Dai Y.J., Zhang W., Zhang W.N., Xiong S.M., Gu Z.H., Wang K.K., Zeng R., Chen Z. (2014). DNMT3A Arg882 mutation drives chronic myelomonocytic leukemia through disturbing gene expression/DNA methylation in hematopoietic cells. Proc. Natl. Acad. Sci. USA.

[92] Ko M., An J., Pastor W.A., Koralov S.B., Rajewsky K., Rao A. (2015). TET proteins and 5-methylcytosine oxidation in hematological cancers. Immunol. Rev..

[93] Lio C.J., Yuita H., Rao A. (2019). Dysregulation of the TET family of epigenetic regulators in lymphoid and myeloid malignancies. Blood.

[94] Ko M., An J., Rao A. (2015). DNA methylation and hydroxymethylation in hematologic differentiation and transformation. Curr. Opin. Cell Biol..

[95] Quivoron C., Couronne L., Della Valle V., Lopez C.K., Plo I., Wagner-Ballon O., Do Cruzeiro M., Delhommeau F., Arnulf B., Stern M.H. (2011). TET2 inactivation results in pleiotropic hematopoietic abnormalities in mouse and is a recurrent event during human lymphomagenesis. Cancer Cell.

[96] Lemonnier F., Couronne L., Parrens M., Jais J.P., Travert M., Lamant L., Tournillac O., Rousset T., Fabiani B., Cairns R.A. (2012). Recurrent TET2 mutations in peripheral T-cell lymphomas correlate with TFH-like features and adverse clinical parameters. Blood.

[97] Odejide O., Weigert O., Lane A.A., Toscano D., Lunning M.A., Kopp N., Kim S., van Bodegom D., Bolla S., Schatz J.H. (2014). A targeted mutational landscape of angioimmunoblastic T-cell lymphoma. Blood.

[98] Sakata-Yanagimoto M., Enami T., Yoshida K., Shiraishi Y., Ishii R., Miyake Y., Muto H., Tsuyama N., Sato-Otsubo A., Okuno Y. (2014). Somatic RHOA mutation in angioimmunoblastic T cell lymphoma. Nat. Genet..

[99] Palomero T., Couronne L., Khiabanian H., Kim M.Y., Ambesi-Impiombato A., Perez-Garcia A., Carpenter Z., Abate F., Allegretta M., Haydu J.E. (2014). Recurrent mutations in epigenetic regulators, RHOA and FYN kinase in peripheral T cell lymphomas. Nat. Genet..

[100] Couronne L., Bastard C., Bernard O.A. (2012). TET2 and DNMT3A mutations in human T-cell lymphoma. N. Engl. J. Med..

[101] Bray J.K., Dawlaty M.M., Verma A., Maitra A. (2021). Roles and Regulations of TET Enzymes in Solid Tumors. Trends Cancer.

[102] Cimmino L., Dawlaty M.M., Ndiaye-Lobry D., Yap Y.S., Bakogianni S., Yu Y., Bhattacharyya S., Shaknovich R., Geng H., Lobry C. (2015). TET1 is a tumor suppressor of hematopoietic malignancy. Nat. Immunol..

[103] Huang H., Jiang X., Li Z., Li Y., Song C.X., He C., Sun M., Chen P., Gurbuxani S., Wang J. (2013). TET1 plays an essential oncogenic role in MLL-rearranged leukemia. Proc. Natl. Acad. Sci. USA.

[104] Huang H., Jiang X., Wang J., Li Y., Song C.X., Chen P., Li S., Gurbuxani S., Arnovitz S., Wang Y. (2016). Identification of MLL-fusion/MYC dash, verticalmiR-26 dash, verticalTET1 signaling circuit in MLL-rearranged leukemia. Cancer Lett..

[105] Bamezai S., Demir D., Pulikkottil A.J., Ciccarone F., Fischbein E., Sinha A., Borga C., Te Kronnie G., Meyer L.H., Mohr F. (2021). TET1 promotes growth of T-cell acute lymphoblastic leukemia and can be antagonized via PARP inhibition. Leukemia.

[106] Poole C.J., Lodh A., Choi J.H., van Riggelen J. (2019). MYC deregulates TET1 and TET2 expression to control global DNA (hydroxy)methylation and gene expression to maintain a neoplastic phenotype in T-ALL. Epigenet. Chromatin.

[107] Wang J., Li F., Ma Z., Yu M., Guo Q., Huang J., Yu W., Wang Y., Jin J. (2018). High Expression of TET1 Predicts Poor Survival in Cytogenetically Normal Acute Myeloid Leukemia From Two Cohorts. eBioMedicine.

[108] Shrestha R., Sakata-Yanagimoto M., Maie K., Oshima M., Ishihara M., Suehara Y., Fukumoto K., Nakajima-Takagi Y., Matsui H., Kato T. (2020). Molecular pathogenesis of progression to myeloid leukemia from TET-insufficient status. Blood Adv..

[109] Kunimoto H., Fukuchi Y., Sakurai M., Sadahira K., Ikeda Y., Okamoto S., Nakajima H. (2012). Tet2 disruption leads to enhanced self-renewal and altered differentiation of fetal liver hematopoietic stem cells. Sci. Rep..

[110] Ko M., Bandukwala H.S., An J., Lamperti E.D., Thompson E.C., Hastie R., Tsangaratou A., Rajewsky K., Koralov S.B., Rao A. (2011). Ten-Eleven-Translocation 2 (TET2) negatively regulates homeostasis and differentiation of hematopoietic stem cells in mice. Proc. Natl. Acad. Sci. USA.

[111] Li Z., Cai X., Cai C.L., Wang J., Zhang W., Petersen B.E., Yang F.C., Xu M. (2011). Deletion of Tet2 in mice leads to dysregulated hematopoietic stem cells and subsequent development of myeloid malignancies. Blood.

[112] Moran-Crusio K., Reavie L., Shih A., Abdel-Wahab O., Ndiaye-Lobry D., Lobry C., Figueroa M.E., Vasanthakumar A., Patel J., Zhao X. (2011). Tet2 loss leads to increased hematopoietic stem cell self-renewal and myeloid transformation. Cancer Cell.

[113] Izzo F., Lee S.C., Poran A., Chaligne R., Gaiti F., Gross B., Murali R.R., Deochand S.D., Ang C., Jones P.W. (2020). DNA methylation disruption reshapes the hematopoietic differentiation landscape. Nat. Genet..

[114] Kaluscha S., Domcke S., Wirbelauer C., Stadler M.B., Durdu S., Burger L., Schubeler D. (2022). Evidence that direct inhibition of transcription factor binding is the prevailing mode of gene and repeat repression by DNA methylation. Nat. Genet..

[115] Yin Y., Morgunova E., Jolma A., Kaasinen E., Sahu B., Khund-Sayeed S., Das P.K., Kivioja T., Dave K., Zhong F. (2017). Impact of cytosine methylation on DNA binding specificities of human transcription factors. Science.

[116] Muto T., Sashida G., Hasegawa N., Nakaseko C., Yokote K., Shimoda K., Iwama A. (2015). Myelodysplastic syndrome with extramedullary erythroid hyperplasia induced by loss of Tet2 in mice. Leuk. Lymphoma.

[117] Zhao Z., Chen S., Zhu X., Pan F., Li R., Zhou Y., Yuan W., Ni H., Yang F.C., Xu M. (2016). The catalytic activity of TET2 is essential for its myeloid malignancy-suppressive function in hematopoietic stem/progenitor cells. Leukemia.

[118] An J., Gonzalez-Avalos E., Chawla A., Jeong M., Lopez-Moyado I.F., Li W., Goodell M.A., Chavez L., Ko M., Rao A. (2015). Acute loss of TET function results in aggressive myeloid cancer in mice. Nat. Commun..

[119] Ito K., Lee J., Chrysanthou S., Zhao Y., Josephs K., Sato H., Teruya-Feldstein J., Zheng D., Dawlaty M.M., Ito K. (2019). Non-catalytic Roles of Tet2 Are Essential to Regulate Hematopoietic Stem and Progenitor Cell Homeostasis. Cell Rep..

[120] Muto H., Sakata-Yanagimoto M., Nagae G., Shiozawa Y., Miyake Y., Yoshida K., Enami T., Kamada Y., Kato T., Uchida K. (2014). Reduced TET2 function leads to T-cell lymphoma with follicular helper T-cell-like features in mice. Blood Cancer J..

[121] Dominguez P.M., Ghamlouch H., Rosikiewicz W., Kumar P., Beguelin W., Fontan L., Rivas M.A., Pawlikowska P., Armand M., Mouly E. (2018). TET2 Deficiency Causes Germinal Center Hyperplasia, Impairs Plasma Cell Differentiation, and Promotes B-cell Lymphomagenesis. Cancer Discov..

[122] Rosikiewicz W., Chen X., Dominguez P.M., Ghamlouch H., Aoufouchi S., Bernard O.A., Melnick A., Li S. (2020). TET2 deficiency reprograms the germinal center B cell epigenome and silences genes linked to lymphomagenesis. Sci. Adv..

[123] Mouly E., Ghamlouch H., Della-Valle V., Scourzic L., Quivoron C., Roos-Weil D., Pawlikowska P., Saada V., Diop M.K., Lopez C.K. (2018). B-cell tumor development in Tet2-deficient mice. Blood Adv..

[124] Zhao Z., Chen L., Dawlaty M.M., Pan F., Weeks O., Zhou Y., Cao Z., Shi H., Wang J., Lin L. (2015). Combined Loss of Tet1 and Tet2 Promotes B Cell, but Not Myeloid Malignancies, in Mice. Cell Rep..

[125] Shukla V., Samaniego-Castruita D., Dong Z., Gonzalez-Avalos E., Yan Q., Sarma K., Rao A. (2022). TET deficiency perturbs mature B cell homeostasis and promotes oncogenesis associated with accumulation of G-quadruplex and R-loop structures. Nat. Immunol..

[126] Olofsen P.A., Fatrai S., van Strien P.M.H., Obenauer J.C., de Looper H.W.J., Hoogenboezem R.M., Erpelinck-Verschueren C.A.J., Vermeulen M., Roovers O., Haferlach T. (2020). Malignant Transformation Involving CXXC4 Mutations Identified in a Leukemic Progression Model of Severe Congenital Neutropenia. Cell Rep. Med..

[127] Ko M., An J., Bandukwala H.S., Chavez L., Aijo T., Pastor W.A., Segal M.F., Li H., Koh K.P., Lahdesmaki H. (2013). Modulation of TET2 expression and 5-methylcytosine oxidation by the CXXC domain protein IDAX. Nature.

[128] Shih A.H., Abdel-Wahab O., Patel J.P., Levine R.L. (2012). The role of mutations in epigenetic regulators in myeloid malignancies. Nat. Rev. Cancer.

[129] Tefferi A. (2011). Mutations galore in myeloproliferative neoplasms: Would the real Spartacus please stand up?. Leukemia.

[130] Shih A.H., Jiang Y., Meydan C., Shank K., Pandey S., Barreyro L., Antony-Debre I., Viale A., Socci N., Sun Y. (2015). Mutational cooperativity linked to combinatorial epigenetic gain of function in acute myeloid leukemia. Cancer Cell.

[131] Abdel-Wahab O., Gao J., Adli M., Dey A., Trimarchi T., Chung Y.R., Kuscu C., Hricik T., Ndiaye-Lobry D., Lafave L.M. (2013). Deletion of Asxl1 results in myelodysplasia and severe developmental defects in vivo. J. Exp. Med..

[132] Kameda T., Shide K., Yamaji T., Kamiunten A., Sekine M., Taniguchi Y., Hidaka T., Kubuki Y., Shimoda H., Marutsuka K. (2015). Loss of TET2 has dual roles in murine myeloproliferative neoplasms: Disease sustainer and disease accelerator. Blood.

[133] Muto T., Sashida G., Oshima M., Wendt G.R., Mochizuki-Kashio M., Nagata Y., Sanada M., Miyagi S., Saraya A., Kamio A. (2013). Concurrent loss of Ezh2 and Tet2 cooperates in the pathogenesis of myelodysplastic disorders. J. Exp. Med..

[134] Kunimoto H., Meydan C., Nazir A., Whitfield J., Shank K., Rapaport F., Maher R., Pronier E., Meyer S.C., Garrett-Bakelman F.E. (2018). Cooperative Epigenetic Remodeling by TET2 Loss and NRAS Mutation Drives Myeloid Transformation and MEK Inhibitor Sensitivity. Cancer Cell.

[135] Palam L.R., Mali R.S., Ramdas B., Srivatsan S.N., Visconte V., Tiu R.V., Vanhaesebroeck B., Roers A., Gerbaulet A., Xu M. (2018). Loss of epigenetic regulator TET2 and oncogenic KIT regulate myeloid cell transformation via PI3K pathway. JCI Insight.

[136] Zang S., Li J., Yang H., Zeng H., Han W., Zhang J., Lee M., Moczygemba M., Isgandarova S., Yang Y. (2017). Mutations in 5-methylcytosine oxidase TET2 and RhoA cooperatively disrupt T cell homeostasis. J. Clin. Investig..

[137] Scourzic L., Couronne L., Pedersen M.T., Della Valle V., Diop M., Mylonas E., Calvo J., Mouly E., Lopez C.K., Martin N. (2016). DNMT3A(R882H) mutant and Tet2 inactivation cooperate in the deregulation of DNA methylation control to induce lymphoid malignancies in mice. Leukemia.

[138] Xu J.J., Chalk A.M., Wall M., Langdon W.Y., Smeets M.F., Walkley C.R. (2022). Srsf2^P95H/+^ co-operates with loss of TET2 to promote myeloid bias and initiate a chronic myelomonocytic leukemia-like disease in mice. Leukemia.

[139] Rasmussen K.D., Jia G., Johansen J.V., Pedersen M.T., Rapin N., Bagger F.O., Porse B.T., Bernard O.A., Christensen J., Helin K. (2015). Loss of TET2 in hematopoietic cells leads to DNA hypermethylation of active enhancers and induction of leukemogenesis. Genes Dev..

[140] Zhang X., Su J., Jeong M., Ko M., Huang Y., Park H.J., Guzman A., Lei Y., Huang Y.H., Rao A. (2016). DNMT3A and TET2 compete and cooperate to repress lineage-specific transcription factors in hematopoietic stem cells. Nat. Genet..

[141] Mendez-Ferrer S., Bonnet D., Steensma D.P., Hasserjian R.P., Ghobrial I.M., Gribben J.G., Andreeff M., Krause D.S. (2020). Bone marrow niches in haematological malignancies. Nat. Rev. Cancer.

[142] Cai Z., Kotzin J.J., Ramdas B., Chen S., Nelanuthala S., Palam L.R., Pandey R., Mali R.S., Liu Y., Kelley M.R. (2018). Inhibition of Inflammatory Signaling in Tet2 Mutant Preleukemic Cells Mitigates Stress-Induced Abnormalities and Clonal Hematopoiesis. Cell Stem Cell.

[143] Cai Z., Lu X., Zhang C., Nelanuthala S., Aguilera F., Hadley A., Ramdas B., Fang F., Nephew K., Kotzin J.J. (2021). Hyperglycemia cooperates with *Tet2* heterozygosity to induce leukemia driven by proinflammatory cytokine-induced lncRNA *Morrbid*. J. Clin. Investig..

[144] Abegunde S.O., Buckstein R., Wells R.A., Rauh M.J. (2018). An inflammatory environment containing TNFalpha favors Tet2-mutant clonal hematopoiesis. Exp. Hematol..

[145] Meisel M., Hinterleitner R., Pacis A., Chen L., Earley Z.M., Mayassi T., Pierre J.F., Ernest J.D., Galipeau H.J., Thuille N. (2018). Microbial signals drive pre-leukaemic myeloproliferation in a Tet2-deficient host. Nature.

[146] Zeng H., He H., Guo L., Li J., Lee M., Han W., Guzman A.G., Zang S., Zhou Y., Zhang X. (2019). Antibiotic treatment ameliorates Ten-eleven translocation 2 (TET2) loss-of-function associated hematological malignancies. Cancer Lett..

[147] Zhang Q., Zhao K., Shen Q., Han Y., Gu Y., Li X., Zhao D., Liu Y., Wang C., Zhang X. (2015). Tet2 is required to resolve inflammation by recruiting Hdac2 to specifically repress IL-6. Nature.

[148] Cull A.H., Snetsinger B., Buckstein R., Wells R.A., Rauh M.J. (2017). Tet2 restrains inflammatory gene expression in macrophages. Exp. Hematol..

